# The Effect of Heparin-Grafted Chitosan-Cellulose Composite Microspheres on the Removal of Endotoxins and Circulating Histones in a Septic Rabbit Model: An In Vivo Study

**DOI:** 10.3390/biomedicines14030661

**Published:** 2026-03-14

**Authors:** Luojia Jiang, Ying Li, Fang Wan, Yi Su, Meixian Lei, Yupei Li, Haibo Xu

**Affiliations:** 1 Department of Nephrology, Jiujiang City Key Laboratory of Cell Therapy, Jiujiang First People’s Hospital, Jiujiang 332000, China; jiangluojia3693@sina.com; 2 Department of General Surgery, Jiujiang City Key Laboratory of Cell Therapy, Jiujiang First People’s Hospital, Jiujiang 332000, China; kathy160527@163.com; 3 Department of Endocrinology, Jiujiang City Key Laboratory of Cell Therapy, Jiujiang First People’s Hospital, Jiujiang 332000, China; wfang0904@163.com; 4 Department of Anesthesiology Jiujiang City Key Laboratory of Cell Therapy, Jiujiang First People’s Hospital, Jiujiang 332000, China; suyiweiei@163.com; 5 Department of Cardiovascular Medicine, Jiujiang City Key Laboratory of Cell Therapy, Jiujiang First People’s Hospital, Jiujiang 332000, China; leimeixian@163.com; 6 Department of Nephrology, Kidney Research Institute, West China Hospital of Sichuan University, Chengdu 610041, China; 7 Department of Hepatology, Jiujiang City Key Laboratory of Cell Therapy, Jiujiang First People’s Hospital, Jiujiang 332000, China

**Keywords:** sepsis, hemoperfusion, endotoxin, histone, hyperinflammation

## Abstract

**Background/Objectives:** The strategy of targeting endotoxins and circulating histones to alleviate excessive inflammation and tissue damage has been proposed as an important immunoregulatory strategy against sepsis. However, the development of a multifunctional hemoperfusion adsorber that simultaneously removes endotoxins and histones remains an unmet clinical need in sepsis management. **Methods**: We synthesized chitosan-cellulose composite (CSCE) microspheres utilizing phase inversion technology, while heparin-grafted chitosan-cellulose composite (CSCEHEP) microspheres were developed by grafting heparin onto CSCE microspheres through the carbodiimide coupling method. In our experimental design, we allocated healthy New Zealand rabbits to four distinct groups: a healthy control group, a lipopolysaccharides (LPS) group, a CSCE group, and a CSCEHEP group. Following the administration of LPS for 12 h, septic rabbits underwent extracorporeal hemoperfusion with either CSCE or CSCEHEP microspheres for a duration of 6 h, notably without the inclusion of heparin in the blood circuits. Post-hemoperfusion, we conducted an analysis of thrombus formation and total protein adsorption on the column. Concurrently, blood samples were collected from the venous side to evaluate inflammatory cytokine concentrations, liver and kidney function levels, LPS levels, the histone presence, and to perform histopathological assessments of liver and kidney injury. **Results**: Our in vivo experiments demonstrated that CSCEHEP microspheres for extracorporeal circulation could achieve a 6 h hemoperfusion session in septic rabbits without the need for continuous anticoagulation with heparin. A CSCEHEP column turns into a very light-red color (almost the original white) and light contamination or clotting was observed after the 6 h hemoperfusion. Moreover, CSCEHEP microspheres effectively reduced the concentration levels of leukocyte, serum IL-6 and TNF-α, mitigated pathological damage to the liver and kidneys, and removed over 56.7% of LPS and nearly 58.6% of histone H3 from the blood of septic rabbits during hemoperfusion. **Conclusions:** Hemoperfusion utilizing CSCEHEP microspheres exhibits excellent self-anticoagulation capabilities, remarkable anti-inflammatory performance, efficient endotoxin adsorption and histone antagonism properties, rendering it both effective and safe for use in septic rabbits.

## 1. Introduction

Sepsis is characterized by an imbalance in the immune response triggered by bacterial, viral, or fungal infections in the host, leading to life-threatening multiple organ dysfunction syndrome (MODS) [1]. Sepsis is a prevalent and severe complication among critically ill patients, marked by high incidence and mortality rates, and imposes a substantial economic burden on healthcare systems globally. Recent epidemiological data from mainland China indicate that the incidence of sepsis is approximately 20.6%, with a 90-day mortality rate of 35.5% and a median medical expenditure reaching up to 50,000 RMB [2]. Current therapeutic approaches for sepsis primarily encompass anti-infective measures, fluid resuscitation, administration of vasopressors, and organ support therapies. However, traditional treatment modalities fail to effectively modulate the immune dysfunction observed in septic patients, thereby not significantly reducing sepsis-related mortality [3]. Therefore, it is of great significance to search for new therapeutic targets for immunoregulatory therapy in sepsis patients and to explore more effective therapeutic regimens.

“Cytokine storm” is a key factor in the occurrence of sepsis-induced MODS, linked to an excessive inflammatory response involving white blood cells, endothelial cells, cytokines, and the coagulation system [4]. Pathogen- and damage-associated molecular patterns activate immune cells, leading to the release of inflammatory cytokines such as interleukin (IL)-1β, IL-6, as well as CXC chemokines such as IL-8. This damages endothelial cells and activates the coagulation system, causing a cytokine storm that results in disseminated intravascular coagulation, microcirculation dysfunction, and MODS [5,6]. Wiersinga et al. found that injecting live bacteria or their cell wall components into live animals triggers a strong inflammatory response, while removing these cytokines can prevent MODS and death [7]. Therefore, alleviating the cytokine storm by regulating the immune response may be a key target for treating sepsis.

Endotoxins, lipopolysaccharides from Gram-negative bacteria, are released upon cell death and recognized by receptors like Toll-like receptor 4, activating signaling pathways [8]. On the one hand, these endotoxins induce a marked increase in inflammatory mediators, including IL-6, IL-8, and endothelial cell adhesion molecules such as intercellular adhesion molecule-1, vascular cell adhesion molecule-1 and E-selectin protein, through the mitogen activated protein kinase and NF-κB pathways. On the other hand, endotoxins contribute to endothelial dysfunction through the nicotinamide adenine dinucleotide phosphate oxidase/reactive oxygen species/endothelial nitric oxide synthase pathway [9].

In recent years, circulating histones have been identified as a novel class of damage-associated molecular patterns. Our previous research demonstrated that circulating histones can mediate excessive inflammatory responses and contribute to organ dysfunction in patients with sepsis [10]. Histones have a molecular weight ranging from approximately 11 to 21 kDa and possess a primary structure abundant in basic amino acids, such as arginine and lysine, which confers a positive charge in bodily fluids [11]. Under normal physiological conditions, histones are primarily involved in chromatin assembly and the regulation of gene transcription. During sepsis, however, inflammatory mediators activate innate immune cells, including neutrophils, macrophages, and monocytes, resulting in a reduced adhesion capacity of histones to immune cells. This reduction leads to the detachment of histones from the cell surface and the formation of circulating histones [12]. Studies have indicated that the concentration of circulating histones in septic patients is significantly elevated compared to healthy individuals (63.5 μg/mL versus 1.5 μg/mL) and is positively correlated with both disease severity and mortality [13]. Our study also revealed that circulating histones can activate immune cells, initiate a “cytokine storm,” and subsequently trigger the activation of the coagulation system and damage to endothelial cells, ultimately resulting in MODS. Various anionic drugs, including heparin, recombinant protein C, and sialic acid, can mitigate the cytotoxic effects of histones by binding to the positively charged histones, thereby inhibiting tissue neutrophil infiltration and inflammatory exudation induced by histones, and alleviating organ damage in sepsis [14]. To date, an increasing number of studies have begun to identify circulating histones as critical immune regulatory factors in the cytokine storm associated with sepsis [15,16,17].

Chitosan is a naturally occurring polysaccharide derived from the deacetylation of chitin, characterized by its high storage capacity and excellent biocompatibility [18]. The molecular structure of chitosan is rich in amino groups and becomes protonated, facilitating the adsorption of endotoxins through electrostatic interactions [19]. In our previous work, we developed self-anticoagulant heparin-grafted chitosan/cellulose composite (CSCEHEP) microspheres for simultaneous adsorption of endotoxin and circulating histones. Our findings demonstrated that the obtained CSCEHEP microspheres significantly prolonged plasma coagulation time while maintaining excellent hemocompatibility, owing to the heparin coating. In vitro, CSCEHEP microspheres exhibited considerable adsorption capacities of 251.0 EU/g for endotoxin, with an adsorption clearance ratio of 81.8%, and 288.6 μg/g for histones, with an adsorption clearance ratio of 62.8%. Additionally, CSCEHEP microspheres effectively adsorbed histones in whole blood and counteracted histone-induced endothelial and kidney epithelial cytotoxicity, erythrocyte fragility, and thrombocytopenia [20]. In this context, the present study seeks to examine the effectiveness of heparin-free hemoperfusion utilizing CSCEHEP microspheres in an LPS-induced septic rabbit model. This investigation focuses on assessing the adsorption efficiency of endotoxins and histones, as well as evaluating the anti-inflammatory and organ-protective properties of CSCEHEP microspheres.

## 2. Materials and Methods

### 2.1. Materials

Chitosan (CS, viscosity: 100–200 mPa·s, deacetylation degree ≥ 95%) powder, α-Cellulose (CE, 90 μm, AR), heparin sodium salt (185 USP units/mg), urea (AR), lithium hydroxide (LiOH, AR), potassium hydroxide (KOH, ACS), and toluidine bule (AR) were purchased from Shanghai Aladdin Biochemical Technology Co., Ltd. (Shanghai, China). 1-Ethyl-3-(3-dimethylaminopropyl) carbodiimide hydrochloride (EDC, 98%) was purchased from McLean Chemical Reagent Co., Ltd. (Shanghai, China). LPS from *Escherichia coli 055:B5*, rabbit IL-6 ELISA Kit, rabbit TNF-α ELISA Kit were purchased from Biyotime Biotechnology Co., Ltd. (Shanghai, China). Rabbit histone H3 ELISA reagent and rabbit LPS ELISA reagent were purchased from Shanghai Renjie Biotechnology Co., Ltd. (Shanghai, China). New Zealand white rabbits were purchased from Nanchang Yongrun Technology Co., Ltd. (Nanchang, China). Measurements of serum bilirubin, aspartate aminotransferase, alanine aminotransferase, urea nitrogen and creatinine were conducted at the Animal Experiment Center of Jiujiang University Affiliated Hospital.

### 2.2. Preparation of CSCE and CSCEHEP Microspheres

**Figure 1:**
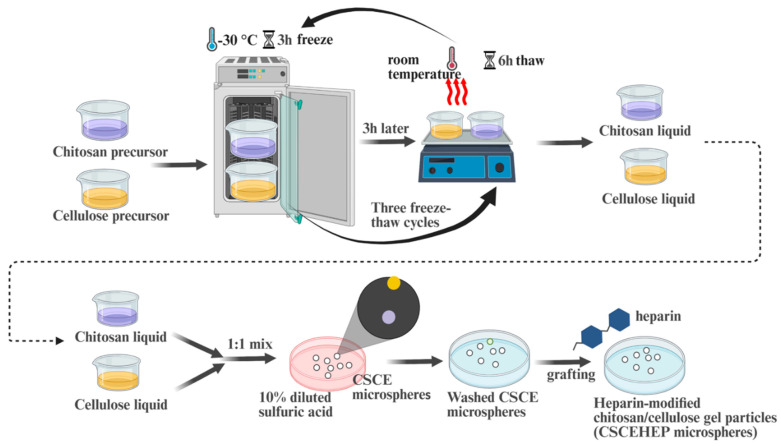
A schematic diagram of the preparation of CSCEHEP microspheres.

According to our previous work [20], the preparation process of CSCE and CSCEHEP microspheres can be found in Figure 1. First, CS and CE solutions (dissolved in 4 wt.% alkaline-urea system) were frozen at −30 °C for 4 h and completely thawed at room temperature. The CS and CE solutions were then mixed in a 1:1 mass ratio and stirred vigorously after three freeze-thaw cycles. Then, CSCE microspheres were obtained by dropping the mixed CE/CS solution uniformly into a 10 wt.% diluted sulfuric acid solution using a syringe needle (28 G). CSCE microspheres were repeatedly cleaned with DI water and stored in DI water. Afterwards, a carbodiimide coupling method was used to prepare CSCEHEP microspheres. In detail, a certain quality heparin sodium salt, CSCE and EDC were reacted in 100 mL of 4-morpholineethanesulfonic acid buffer solution (0.1 M) at room temperature for 24 h. The CSCEHEP microspheres were washed with DI water and then stored in PBS.

### 2.3. Efficacy of Extracorporeal Hemoperfusion with CSCEHEP in a Septic Rabbit Model

The experiments were approved by the Ethics Committee of Jiujiang First People’s Hospital (JJSDYRMYY-YXLL-2023-129). The experimental rabbits were provided by Nanchang Yongrun Technology Co., Ltd. (Nanchang, China). Healthy New Zealand white male rabbits (~2.5 kg, age 3–4 months) were randomly housed two per cage, and could access water and chow free at room temperature. Animals were randomly assigned to different groups via an electronic randomization system, and all subsequent procedures and analyses were conducted under blinding conditions. We randomly divided 24 rabbits into four groups, including a healthy control group, an LPS group, a CSCE group, and a CSCEHEP group, with each group comprising 6 rabbits. Trained personnel performed all animal handling and surgeries. The healthy control group did not undergo any surgical operation, only anesthesia treatment. In the LPS group, *Escherichia coli* LPS was intravenously injected into the ear vein at a dosage of 500 μg·kg^−1^. There was careful monitoring of vital signs like the oxygen saturation, pulse rate, respiratory rate, and blood pressure while performing the procedure. After 12 h of LPS injection, the rabbits showed symptoms such as fever, lethargy, and poor appetite, confirming the successful establishment of a septic rabbit model. Before the start of hemoperfusion, anesthesia was induced in the rabbits through an intravenous injection of 3% pentobarbital sodium at a dosage of 1 mL·kg^−1^. Subsequently, the right femoral artery and vein were isolated and secured individually using indwelling needles (24 G) to facilitate connection to a custom-made CSCE or CSCEHEP hemoperfusion column. The blood circuit was prepared using a 30 G blood collection needle tubing, with connections secured by heparin caps. After the rabbits were intravenously injected with LPS and maintained for 12 h at room temperature, extracorporeal hemoperfusion was performed using a home-made column (7.5 mL) packed with either CSCE or CSCEHEP microspheres. Blood was circulated from the femoral artery to the femoral vein at a flow rate of 5 mL/min, which was controlled by a Baoding Lange BT100-3J peristaltic pump. Venous blood samples were collected for the analysis of LPS, histones, inflammatory cytokines, and additional parameters at different time points. After 6 h of hemoperfusion, the blood in the column was returned to the rabbit’s body using physiological saline and the surgical incision was carefully sutured. Proteins on the hemoperfusion column were eluted using the pH gradient elution method [21,22] and quantified using the BCA method. The thrombus and total protein adsorption on the column were studied and histopathological injury of the liver and kidneys was assessed within 24 h after extracorporeal hemoperfusion. After the experiment, we administered a high dose of anesthesia to the rabbits to ensure rapid loss of consciousness and humane euthanasia. Subsequently, the remains were cremated by certified pet cremation services.

### 2.4. Statistical Analysis

In this study, either one-way or two-way ANOVA were employed to compare the differences in measured outcomes among different groups. Experimental data are expressed as mean ± standard deviation. No statistical method was used to determine whether the data met assumptions of the statistical approach. A *p*-value less than 0.05 was considered to indicate a statistically significant difference. Statistical analyses were performed using the GraphPad Prism 8.0.1 software.

## 3. Results

### 3.1. Chemical Characterization of CSCEHEP Microspheres

Our previous research has successfully characterized and identified the chemical features of CSCEHEP microspheres. Firstly, the pore sizes of the CSCEHEP microspheres predominantly range from 2 to 10 nm, categorizing them as mesoporous. Secondly, the heparin leakage from the CSCEHEP microspheres was negligible during short-term storage of 28 days. Thirdly, the deformation of CSCEHEP microspheres under a maximum transmembrane pressure of 66.7 kPa (500 mmHg) is within an acceptable range, fulfilling clinical requirements for hemoperfusion adsorbents. Lastly, in SEM images, the CSCEHEP microspheres feature a loose internal porous structure and a dense outer shell, characterized by high porosity and a specific surface area of 24.1972 m^2^/g.

### 3.2. Self-Anticoagulation Property of the Home-Made CSCEHEP Hemoperfusion Column

**Figure 2:**
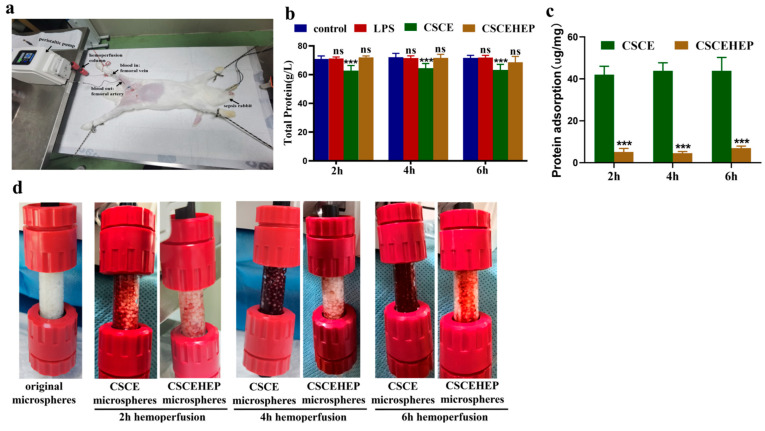
A schematic diagram of a hemoperfusion session with the home-made CSCEHEP column in septic rabbits. (**a**) Photographs of the hemoperfusion of septic rabbits; (**b**) an assessment of plasma total protein levels; (**c**) circulating protein adsorption amount; (**d**) filter coagulation display after hemoperfusion (n = 6). Data are expressed as the means ± SD. ns, *p* > 0.05; ***, *p* < 0.001 compared to control group (n = 6).

To better meet the needs of hemoperfusion, we redesigned the adsorbents used in hemoperfusion devices by utilizing CSCE or CSCEHEP microspheres as the substrate. We filled a sterile chromatography column measuring 10 mm × 10 mm with 20 mg of sterilized CSCE or CSCEHEP microspheres, occupying 95% of the column’s space, and primed it with physiological saline to remove air. Additionally, the CSCE and CSCEHEP hemoperfusion column was pre-circulated with 20 mL physiological saline containing 25% sodium heparin for 30 min (Figure 2a). Meanwhile, sham-hemoperfusion was carried out using a column filled with glass beads as the LPS group. We assessed the impact of each experimental group on the plasma total protein levels. Relative to the control group, the plasma total protein concentration was significantly decreased in the CSCE microsphere group. In contrast, no significant reduction in plasma total protein was observed in either the LPS group or the CSCEHEP group (*p*-values all <0.001) (Figure 2b). We detected a total protein adsorption capacity of 42.03 ± 3.98 μg·mg^−1^ on the CSCE column, exceeding 8.48-fold of the CSCEHEP column (5.12 ± 1.70 μg·mg^−1^) in 2 h hemoperfusion. As the duration of hemoperfusion was extended to 6 h, a light significant increase was observed in the adsorption capacity of CSCEHEP microspheres compared to 2 h hemoperfusion of CSCEHEP microspheres (8.62 ± 2.85 μg·mg^−1^ vs. 5.12 ± 1.70 μg·mg^−1^, *p*-values = 0.0272) (Figure 2c). As depicted in Figure 2d, the CSCE column turned to dark red following the 2 h hemoperfusion (original color: white), indicating significant coagulation and thrombogenesis within the CSCE hemoperfusion column. In contrast, the CSCEHEP microspheres column displayed a very light-red color, nearly retaining its original white color, with no evident contamination or clotting observed after the 2 h hemoperfusion. Following 2 and 4 h of hemoperfusion, the CSCEHEP microsphere column exhibited negligible clotting. A minimal amount of clotting was observed only after 6 h of hemoperfusion. In contrast, the CSCE microsphere column exhibited Grade 2 coagulation after 2 h of hemoperfusion, which required the administration of additional heparin to successfully complete the 6 h hemoperfusion process. These findings demonstrate that CSCEHEP microspheres possess excellent self-anticoagulation properties in vivo, attributed to the heparin coating.

### 3.3. Effect of Hemoperfusion with CSCEHEP Microspheres on Blood Cells

**Figure 3:**
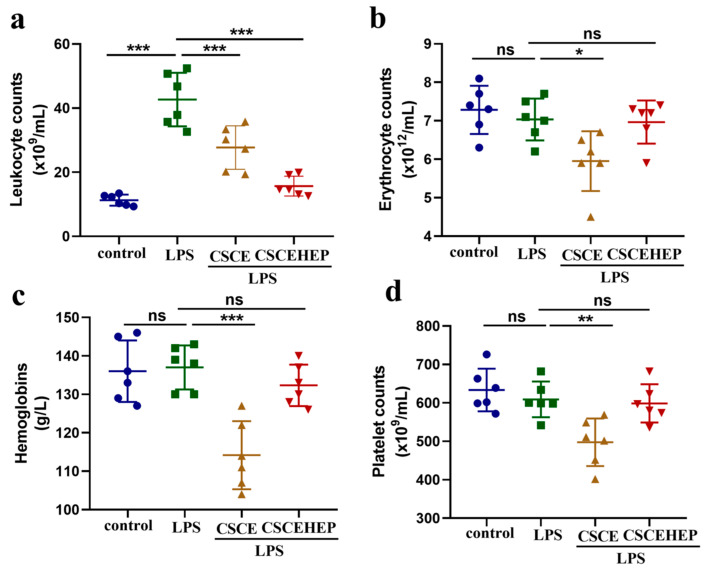
Effect of hemoperfusion with CSCEHEP microspheres on blood cells. (**a**–**d**) Blood routine sequentially including leukocytes, erythrocytes, hemoglobin and platelets for hemoperfusion by CSCE and CSCEHEP microspheres. Data are expressed as the means ± SD. ns, *p* > 0.05; *, *p* < 0.05; **, *p* < 0.01; ***, *p* < 0.001 (n = 6).

In the course of our animal experiments, we initially assessed the effects of CSCEHEP microspheres on hematological parameters. As shown in Figure 3a, compared to the control group, all the sepsis rabbits suffered from severe inflammatory reactions after the LPS injection and the number of leukocyte counts increased remarkably by nearly 73% (*p*-values < 0.001). In comparison to the LPS group, both the CSCE and CSCEHEP groups demonstrated a statistically significant reduction in white blood cell counts, with the reduction being more pronounced in the CSCEHEP group (*p*-values < 0.001), which revealed that CSCEHEP microspheres eliminated the LPS-induced inflammatory reaction during the hemoperfusion session. Figure 3b,c demonstrates that the erythrocyte counts and hemoglobin levels in the CSCE group showed a significant decrease compared with the LPS group due to local thrombus formation (*p*-values = 0.036, *p*-values < 0.001). However, there was no significant difference in erythrocyte counts and hemoglobin levels between the CSCEHEP group and the LPS group (*p*-values = 0.998, *p*-values = 0.675), indicating that the heparin layer on CSCEHEP exerts sufficient anticoagulant effect to avoid local thrombus formation. As for the platelet counts, Figure 3d shows that the CSCE group exhibited a significant decrease in platelet counts compared with the LPS group (*p*-values = 0.009), which was also due to the platelet consumption caused by column thrombosis, while the CSCEHEP group showed no significant change in platelet counts (*p*-values = 0.986).

### 3.4. Effect of Hemoperfusion with CSCEHEP Microspheres on Inflammatory Responses

**Figure 4:**
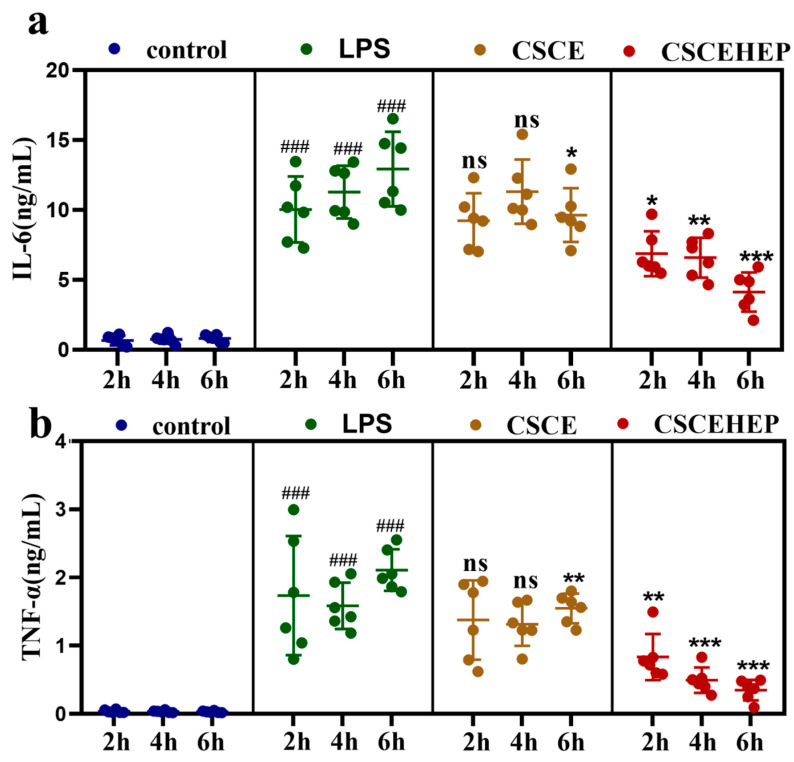
Effect of hemoperfusion with CSCEHEP microspheres on inflammatory factors. (**a**) Serum IL-6 concentration levels. (**b**) Serum TNF-α concentration levels. Data are expressed as means ± SD. ###, *p*-value < 0.05 compared to control group in corresponding time point. ns, *p*-value > 0.05; *, *p*-value < 0.05; **, *p*-value < 0.01; ***, *p*-value < 0.001 compared to LPS group in corresponding time point (n = 6).

Next, the immunoregulatory property of CSCEHEP microspheres in the septic rabbit model was studied, and we chose IL-6 and TNF-α as indicators of the inflammatory response. As shown in Figure 4a,b, compared with the control group, the concentrations of IL-6 and TNF-α in septic rabbits significantly increased after the intravenous injection of LPS for 12 h (*p*-values all < 0.001). Compared to the LPS group after 2 h hemoperfusion, the IL-6 concentration in the CSCEHEP group was significantly reduced (*p*-values = 0.0218). As the duration of hemoperfusion was extended, the IL-6 levels in the CSCEHEP group exhibited a significant further reduction at both the 4 h (*p*-values = 0.002) and 6 h (*p*-values < 0.001) marks when compared to the LPS group. However, the CSCE group showed no significant decrease in IL-6 concentration in the 2 to 4 h hemoperfusion session (*p*-values all > 0.05), apart from 6 h of hemoperfusion (*p*-values < 0.0343). Compared with the LPS group, we can see both the CSCE group and CSCEHEP group showed no significant decrease in TNF-α concentration when 2 h hemoperfusion (*p*-values = 0.604, *p*-values = 0.066). But with the extension of the hemoperfusion time, the TNF-α concentration in the CSCEHEP group significantly decreased at both 4 h (*p*-values < 0.001) and 6 h (*p*-values < 0.001). In the CSCE microsphere group, a particularly meaningful decrease in TNF was observed only at the 6 h hemoperfusion (*p*-values < 0.001). The findings suggest hemoperfusion using CSCEHEP microspheres effectively reduces IL-6 and TNF-α level compared to CSCE microspheres in rabbits with sepsis. Although the anti-inflammatory effect of CSCEHEP microspheres is not markedly pronounced at 2 h post-perfusion, it reaches its maximum efficacy at 4 h and maintains this superior anti-inflammatory effect for up to 6 h.

### 3.5. Effect of Hemoperfusion with CSCEHEP Microspheres on Liver Function and Renal Function

**Figure 5:**
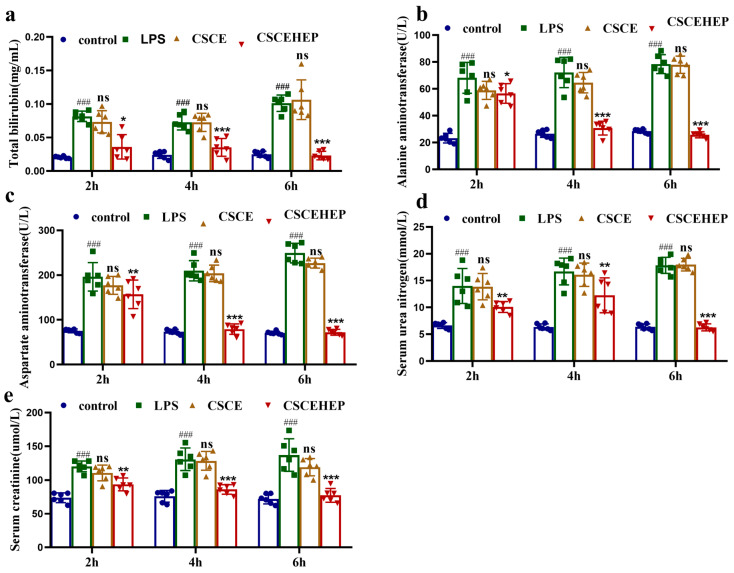
Effects of CSCEHEP microspheres hemoperfusion on liver and kidney function. (**a**) Serum total bilirubin concentration levels; (**b**) serum alanine transaminase concentration levels; (**c**) serum aspartate transaminase concentration levels; (**d**) serum urea nitrogen concentration levels; (**e**) serum creatinine concentration levels. ###, *p*-value < 0.05 compared to control group in corresponding time point. ns, *p*-value > 0.05; *, *p*-value < 0.05; **, *p*-value < 0.01; ***, *p*-value < 0.001 compared to LPS group in corresponding time point (n = 6).

We use serum total bilirubin, aspartate transaminase, and alanine transaminase activities to reflect the dynamic changes of liver function injury in a sepsis rabbit. Serum urea nitrogen and creatinine are used to evaluate changes in renal injury. In the LPS group in 2 h to 6 h hemoperfusion, the total bilirubin, the aspartate transaminase as well as the alanine transaminase level exhibit a statistically significant increase compared to the control group (all *p*-values < 0.001). Compared to the LPS group, the CSCEHEP group exhibit a significant decrease in the aforementioned indicators in the corresponding time period. The decline reaches its apex at 4 h of hemoperfusion, and this maximal effect can be sustained until 6 h of hemoperfusion (all *p*-values < 0.001) (Figure 5a–c). As illustrated in Figure 5d,e, the serum urea nitrogen and creatinine level in the LPS group show a significant increase compared to the control group in the corresponding time period (all *p*-values < 0.001). Both the serum urea nitrogen and the creatinine level in the CSCEHEP group show a significant decrease compared to the LPS group in 2 h hemoperfusion (all *p*-values < 0.01). Following the extension of the hemoperfusion to a duration of 6 h, there is a further reduction in the levels of blood urea nitrogen and serum creatinine observed in the CSCEHEP group (all *p*-values < 0.001).

### 3.6. Effect of Hemoperfusion with CSCEHEP Microspheres on Pathological Damage of Liver and Kidney

**Figure 6:**
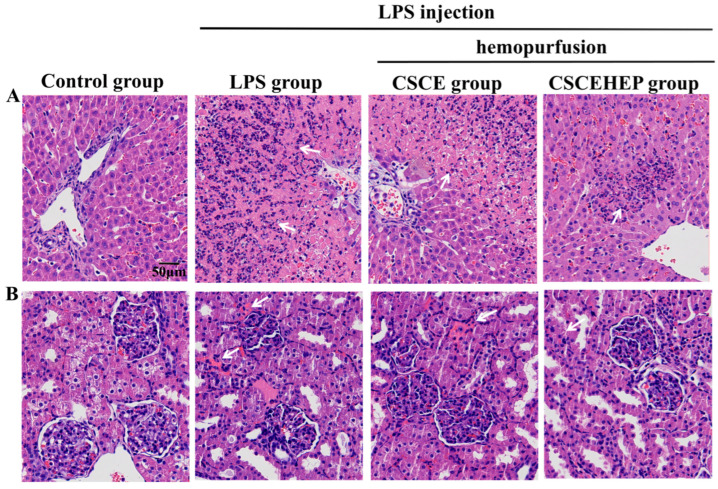
The effect of CSCEHEP microspheres hemoperfusion on the pathology of liver (**A**) and kidney (**B**) tissues. The white arrow indicates the infiltration of inflammatory cells.

We further evaluated the influence of hemoperfusion with CSCEHEP microspheres on the pathological damage of the liver and kidney in septic rabbits. When challenged with LPS, the liver and kidney tissues of rabbits exhibit rapid and severe inflammatory reactions compared to the control group. In contrast, timely hemoperfusion treatment with CSCEHEP microspheres significantly reduces the infiltration of inflammatory cells in liver tissue (as indicated by the white arrow) (Figure 6A). Additionally, in comparison to the control group, the LPS group demonstrates pronounced inflammatory cell infiltration in kidney tissue. Hemoperfusion with CSCEHEP microspheres markedly diminishes inflammation in renal tissue compared to the LPS group (as indicated by the white arrow) (Figure 6B).

### 3.7. Effect of Hemoperfusion with CSCEHEP Microspheres on Serum Levels of LPS and Histone H3

**Figure 7:**
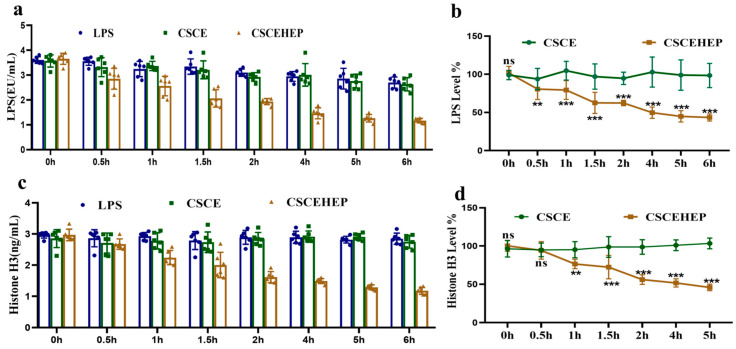
Circulating LPS and histone H3 clearance in septic rabbits of CSCEHEP microspheres hemoperfusion. (**a**) Circulating LPS levels; (**b**) percentage decrease in circulating LPS levels; (**c**) circulating histone H3 levels; (**d**) percentage decrease in circulating histone H3 levels. Data are expressed as means ± SD. ns, *p*-value > 0.05; **, *p*-value < 0.01; ***, *p*-value < 0.001 compared to LPS group in corresponding time point (n = 6).

As shown in Figure 7a,b, the peripheral blood LPS level in the LPS group reached a relatively high level of 3.60 ± 0.13 EU·mL^−1^. After 6 h, the LPS levels in the LPS group decreased to 2.7 ± 0.22 EU·mL^−1^ even in the absence of hemoperfusion. This reduction can be attributed to the body’s intrinsic mechanisms for LPS clearance and neutralization, which include metabolic clearance by the liver, neutralization by plasma proteins, and phagocytic degradation by the immune system. By contrast, in the hemoperfusion group using CSCEHEP microspheres for a duration of 2 h, a progressive decline in the LPS level from 3.10 ± 0.15 EU·mL^−1^ to 1.93 ± 0.12 EU·mL^−1^ (*p*-values < 0.001), and more than 37.7% of the LPS in blood could be removed by the 7.5 mL column (~3.0 g CSCEHEP microsphere). As the duration of hemoperfusion was extended, the level of LPS decreased to 1.46 ± 0.22 EU·mL^−1^ in the CSCEHEP group after 4 h, corresponding to a 50.5% reduction. After 6 h, the LPS level further declined to 1.17 ± 0.09 EU·mL^−1^ in the CSCEHEP group, demonstrating an approximate 56.7% clearance. We can see from Figure 7c,d that the peripheral blood histone H3 level in the LPS group reached a relatively high level of 2.97 ± 0.10 ng·mL^−1^. At 6 h, the histone H3 level slightly declined to 2.85 ± 0.18 ng·mL^−1^ in the LPS group. After 2 h of hemoperfusion, we can see the histone H3 levels significantly decreased to 1.62 ± 0.18 ng·mL^−1^ in the CSCEHEP group (*p*-values < 0.01); nearly 43.9% of histone H3 in the blood could be removed. After 6 h of hemoperfusion, the concentration of histone H3 decreased by 58.6%, resulting in a final concentration of 1.18 ± 0.11 ng·mL^−1^. However, in the CSCE group, there were no significant decreases in either the LPS or the histone H3 level compared to the LPS group during the 6-h hemoperfusion (all *p*-values > 0.05).

## 4. Discussion

The key inflammatory mediators, such as endotoxins and circulating histones, are pivotal in the onset and progression of the “cytokine storm” associated with sepsis. Addressing the issue of immune imbalance in sepsis is critically important for effective treatment; however, this aspect is frequently neglected in clinical settings. With advancements in hemoperfusion technologies, hemoperfusion adsorbents are increasingly employed to eliminate abnormal cytokines, endotoxins, and other inflammatory mediators from the bloodstream of sepsis patients. Despite this, there is a notable lack of hemoperfusion devices specifically designed to remove circulating histones. Currently, the most commonly utilized hemoperfusion adsorbents in clinical practice include polymyxin B (PMX) hemoperfusion columns and the oXiris^®^ membrane. These adsorbents have been shown to decrease endotoxin levels in the blood of sepsis patients to varying extents; however, their impact on improving patient survival rates remains inconclusive. According to Dellinger et al., PMX does not significantly reduce the 28-day mortality rate in sepsis patients with an endotoxin activity assay (EAA) greater than 0.6 [23], and only when the endotoxin load is low (EAA 0.6–0.9) can PMX reduce the short-term mortality rate of patients [24]. The oXiris membrane material is an AN69ST membrane modified with polyethyleneimine cationic polymer, which has triple functions of endotoxin adsorption, cytokine clearance, and kidney replacement [25]. Small sample queue studies have shown that the oXiris membrane can significantly reduce SOFA scores and improve hemodynamics of septic patients [26]. A retrospective study found that the oXiris membrane could reduce SOFA scores faster, but its long-term survival benefit in septic patients is still unknown [27]. In addition, the median filter life of the oXiris membrane is lower (13 h vs. 16 h), and it is more prone to hemofilter coagulation [28], associated with a high dependence on heparin or citrate anticoagulation. CytoSorb^®^ is among the most extensively utilized hemoperfusion devices, capable of reducing the levels of hydrophobic molecules with molecular weights up to 55 kDa [29]. However, there is no evidence indicating that CytoSorb^®^ adsorbents positively impact mortality across various diagnoses, which raises questions about their widespread application in intensive care settings. The efficacy of CytoSorb^®^ in reducing mortality remains uncertain, and studies examining the theoretical foundation of the treatment—specifically, the reduction of blood cytokine levels—have yielded inconsistent results. Notably, CytoSorb^®^ adsorbents have not been shown to significantly decrease IL-6 levels. Honore et al. [30] suggest that while cytokines are removed during hemoperfusion, their levels may remain stable due to the transfer of additional cytokines from the interstitial space to the bloodstream. Even if pro-inflammatory cytokines are adequately removed, another potential reason for the lack of mortality reduction is the non-selective removal of all hydrophobic plasma components with molecular weights up to 55 kDa, which includes anti-inflammatory mediators, hormones, and coagulation factors [31,32]. In conclusion, current hemoperfusion adsorbents encounter several clinical challenges, including limited adsorption efficiency, a significant dependence on heparin or citrate anticoagulation, a narrow range of adsorption targets, and suboptimal blood compatibility. The advancement of domestically produced, high-efficiency endotoxin adsorbents that enhance adsorption capacity while simultaneously addressing the clearance of diverse pathogenic factors—such as inflammatory mediators, pathogenic bacteria, and histones—and incorporating self-anticoagulant properties, is of paramount importance. These developments have the potential to improve the survival prognosis of sepsis patients.

Our previous study indicated that CSCEHEP microspheres demonstrated excellent blood compatibility and self-anticoagulant properties, showing no significant activation of complement, contact or kinin systems, nor causing marked hemolysis, cytopenia or platelet activation. Featuring a loosely porous internal structure with high specific surface area, CSCEHEP microspheres adsorbed 81.8% endotoxin in PBS with an adsorption capacity of 251.0 EU/g. The histone adsorption capacity of CSCEHEP microspheres was 288.6 μg/g in PBS, with a histone clearance ratio of 62.8%. Furthermore, CSCEHEP microspheres effectively adsorbed histones in whole blood and counteracted histone-induced cytotoxicity, erythrocyte osmotic fragility and thrombocytopenia [20]. These results indicate that the heparin layer on CSCE microspheres can still exhibit self-anticoagulant properties, maintain smooth local blood flow in the extracorporeal pipeline during hemoperfusion, and prevent the risk of bleeding caused by traditional systemic heparinization. This greatly avoids the bleeding risk of systemic anticoagulation in critically ill diseases such as sepsis treated with hemoperfusion therapy [33]. However, the LPS and histone clearance rate in the PBS buffer cannot be compared with that of plasma or blood samples due to the combination of plasma proteins and lipoproteins induced to voltage for binding and mask surface charges. To further validate the self-anticoagulation properties of CSCEHEP microspheres, as well as their clearance and adsorption efficiency in immune regulation within animal models, we developed a hemoperfusion device specifically for sepsis rabbit models. Subsequently, we assessed the safety and efficacy of CSCEHEP microspheres in the treatment of sepsis.

In this study, we employed CSCEHEP microspheres for 6 h hemoperfusion treatment in septic rabbits, notably achieving the procedure without the use of heparin. we found that CSCEHEP microspheres could reduce the increase of leukocyte counts induced by LPS in septic rabbits, indicating that they can exert anti-inflammatory effects through certain mechanisms. Chitosan has been confirmed by multiple studies to have significant anti-inflammatory effects, which involve regulating immune responses, inhibiting the release of pro-inflammatory factors, and promoting tissue repair. Wang et al. [34] showed chitosan-based immunomodulatory bio-adhesive hydrogel promoted macrophage polarization toward an M2 phenotype with significantly downregulated pro-inflammatory gene expression, upregulated anti-inflammatory gene expression, and decreased levels of reactive oxygen species. Its primary mechanism involves activating the cGAS-STING signaling pathway in dendritic cells (DCs), which promotes DC maturation in a type I interferon (IFN-I)-dependent manner, ultimately leading to enhanced antigen-specific T helper 1 (Th1) cell responses. Li et al. [35] reported that chitosan upregulated the expression of IL-12 and IL-15 by activating DCs, thereby activating STAT4 and NF-κB signaling pathways in natural killer (NK) cells and promoting NK cells’ survival. CSCE microspheres we synthesized did not result in a significant reduction of IL-6 and TNF-α levels following 2 and 4 h of hemoperfusion. However, a notable decrease in these indicators was observed after 6 h of hemoperfusion. Heparin is not only a classic anticoagulant, but has also been shown to exert anti-inflammatory effects through mechanisms such as inhibiting the release of inflammatory mediators, regulating immune cell function, protecting endothelial cells, and clearing free radicals [36,37,38]. The heparin molecule, forming a stable complex with the cytokine, blocks its binding site I and prevents helices A and D from getting in close contact with IL-6Rα. On the one hand, heparin breaks the cytokine signaling pathway by inhibiting the formation of the IL-6/IL-6Rα complex. On the other hand, binding to the complex IL-6/IL-6Rα, heparin blocks binding site II of IL-6, thus preventing helices A and C from being in a proper position to bind to the gp130 receptor [39]. Heparin alleviates inflammatory disorders by targeting the HMGB-1-associated signaling pathways, which was found to interfere with the HMGB-1/RAGE axis by competitively binding HMGB-1 with extremely high affinity [40]. Moreover, heparin blocks the binding of HMGB-1 to the surface of macrophages and inhibits LPS-induced HMGB-1 amplified inflammatory responses through inhibiting phosphorylation of p38 and extracellular signal-regulated kinase-1/2 [41]. In our study, CSCEHEP microspheres could reduce IL-6 levels in an LPS-induced sepsis rabbit model in 2 h hemoperfusion. Over time, the reduction in IL-6 levels becomes increasingly pronounced, confirming that the CSCEHEP microspheres can stabilize anti-inflammatory effects, which might be attributed to the anti-inflammatory effect of chitosan and heparin components in the CSCEHEP microspheres. Sepsis can rapidly cause tissue damage to multiple organs, leading to liver failure and kidney failure [42]. The application of CSCEHEP microspheres resulted in a significant reduction in serum bilirubin, alanine aminotransferase, aspartate aminotransferase, blood urea nitrogen, and creatinine levels, commencing at 2 h post-initiation of hemoperfusion. This effect was sustained and became more pronounced with prolonged hemoperfusion duration to 6 h. Furthermore, CSCEHEP microspheres can also significantly reduce inflammatory cells in liver and kidney tissues, and improve histopathological damage.

Endotoxins are one of the core targets in the pathophysiological mechanism of sepsis, especially in sepsis associated with Gram-negative bacterial infections. Recent studies have shown that extracellular histones are another novel potential mediator of sepsis induced death [43,44]. Histones are the most abundant proteins in the nucleus, consisting of adaptor histone H1 and core histones H2A, H2B, H3, and H4 [45]. In animal models of sepsis, histones are released into interstitial spaces where they induce local inflammatory cell aggregation, endothelial damage, and organ dysfunction [37,38]. Research has shown that the level of circulating histone H3 is closely associated with coagulation disorder [46]. It follows that evaluating histone H3 levels is not only important for identifying the severity of sepsis, but also for developing histone-targeted therapy strategies. Our study demonstrated a significant elevation in the concentrations of LPS and histone H3 in septic rabbits. With the extension of hemoperfusion duration, the efficacy of CSCEHEP microspheres hemoperfusion in clearing LPS and histone H3 are progressively enhanced. After 2 h of hemoperfusion, the CSCEHEP microspheres hemoperfusion successfully removed over 37.7% of LPS and approximately 43.9% of histone H3 from the bloodstream of septic rabbits. Following a 6 h hemoperfusion session, CSCEHEP microspheres demonstrated an approximate clearance rate of 56.7% for LPS and 58.6% for histones. Simultaneously, no notable protein contamination or coagulation was detected on the CSCEHEP column during 6 h hemoperfusion conducted in the absence of heparin.

Interestingly, we found a significant decrease in the LPS adsorption capacity of CSCE microspheres in the hemoperfusion setting compared to that in PBS. In our previous study, batch adsorption data showed that CSCE microspheres had significantly higher LPS adsorption capacity than CSCEHEP microspheres (577.7 EU/g vs. 250.8 EU/g) [20]. We consider, due to the lack of sustained heparin anticoagulation in hemoperfusion with CSCE microspheres, there was significant plasma protein adsorption and thrombus formation in the CSCE hemoperfusion column (as shown in Figure 2c,d), which may block the LPS binding sites on the surface of CSCE microspheres. This greatly reduced the electrostatic adsorption capacity of chitosan for LPS. However, CSCEHEP microspheres have a self-anticoagulant heparin coating, which makes the adsorption of CSCEHEP microspheres hemoperfusion column proteins and thrombus formation extremely rare. In this regard, the exposed binding sites of LPS on CSCEHEP microspheres can be fully utilized. Therefore, the CSCEHEP microspheres not only achieve local anticoagulation in the hemoperfusion column, but also have good LPS and histone clearance capacity.

Of note, current Surviving Sepsis Campaign guidelines do not endorse the use of hemoperfusion in the management of sepsis, due to its contradictory therapeutic efficacy in existing therapeutic protocols [47]. Nevertheless, numerous studies have also validated the effectiveness of adsorption filters and Polymyxin B endotoxin patients in particular subgroups of sepsis patients. We believe that hemoperfusion therapies are not universally applicable to all sepsis patients. Only specific populations, such as those with an EAA value between 0.6 and 0.9, are appropriate candidates for endotoxin adsorption [24]. Similarly, patients exhibiting elevated levels of endotoxins and histones may benefit from simultaneous removal of endotoxin and histones by our CSCEHEP microspheres. This underscores our recommendation for tailoring hemoperfusion therapy to the specific immune subtypes of patients with sepsis.

## 5. Limitations of the Study

This study is subject to several limitations. Firstly, this study did not evaluate the mortality of rabbits with sepsis in each group, thereby precluding an assessment of the final prognosis for rabbits with sepsis treated with CSCEHEP microspheres. Secondly, the specificity of peripheral blood histone H3 concentration as a marker for sepsis requires further investigation, as elevated levels of circulating histone H3 are also observed in non-infectious conditions such as trauma and pancreatitis. Thirdly, the LPS injection model does not fully replicate the complex and dynamic pathophysiology of a live bacterial infection in human sepsis. We will explore a bolus LPS injection model to ensure the stability of the LPS intravenous injection.

## 6. Conclusions

In conclusion, the CSCEHEP microspheres we have developed exhibit effective self-anticoagulation properties and can achieve 6 h heparin-free hemoperfusion therapy in septic rabbits. This advancement addresses the challenges of coagulation dysfunction and elevated bleeding risk in patients with sepsis or those who are critically ill. CSCEHEP microspheres have been shown to effectively mitigate the systemic inflammatory response in septic rabbits. This is evidenced by a significant reduction in serum leukocyte count, interleukin-6 (IL-6), and tumor necrosis factor-alpha (TNF-α) levels. Additionally, there is marked improvement in the liver and kidney function, accompanied by a substantial decrease in tissue inflammatory cell infiltration. These findings suggest that CSCEHEP microspheres may alleviate the systemic manifestations of sepsis and prevent organ failure resulting from dysregulated inflammatory responses. Furthermore, CSCEHEP microspheres demonstrated the capability to adsorb 56.7% of LPS and effectively removed approximately 58.6% of histone H3 in septic rabbits, achieving dual-targeted adsorption of LPS and histone from the pathogenic source of sepsis. Therefore, CSCEHEP microspheres are promising hemoperfusion adsorbents with simultaneous endotoxin and histone adsorption for future extracorporeal hemoperfusion in sepsis patients.

## Data Availability

The data supporting the results of this study are available from the corresponding author upon reasonable request.

## References

[1] Shankar-Hari M., Phillips G.S., Levy M.L., Seymour C.W., Liu V.X., Deutschman C.S., Angus D.C., Rubenfeld G.D., Singer M., for the Sepsis Definitions Task Force (2016). Developing a new definition and assessing new clinical criteria for septic shock: For the third international consensus definitions for sepsis and septic shock (sepsis-3). JAMA.

[2] Xie J., Wang H., Kang Y., Zhou L., Liu Z., Qin B., Ma X., Cao X., Chen D., Lu W. (2020). The epidemiology of sepsis in chinese icus: A national cross-sectional survey. Crit. Care Med..

[3] Song C., Xu J., Gao C., Zhang W., Fang X., Shang Y. (2022). Nanomaterials targeting macrophages in sepsis: A promising approach for sepsis management. Front. Immunol..

[4] Iba T., Levi M., Levy J.H. (2022). Intracellular communication and immunothrombosis in sepsis. J. Thromb. Haemost..

[5] Moriyama K., Nishida O. (2021). Targeting cytokines, pathogen-associated molecular patterns, and damage-associated molecular patterns in sepsis via blood purification. Int. J. Mol. Sci..

[6] Angus D.C., van der Poll T. (2013). Severe sepsis and septic shock. N. Engl. J. Med..

[7] Wiersinga W.J., Leopold S.J., Cranendonk D.R., van der Poll T. (2014). Host innate immune responses to sepsis. Virulence.

[8] Thon P., Rump K., Knorr A., Dyck B., Ziehe D., Unterberg M., Nowak H., Bergmann L., Wolf A., Bazzi M. (2022). The distinctive activation of toll-like receptor 4 in human samples with sepsis. Cells.

[9] Grylls A., Seidler K., Neil J. (2021). Link between microbiota and hypertension: Focus on lps/tlr4 pathway in endothelial dysfunction and vascular inflammation, and therapeutic implication of probiotics. Biomed. Pharmacother..

[10] Li Y., Wan D., Luo X., Song T., Wang Y., Yu Q., Jiang L., Liao R., Zhao W., Su B. (2021). Circulating histones in sepsis: Potential outcome predictors and therapeutic targets. Front. Immunol..

[11] Singh R.K., Paik J., Gunjan A. (2009). Generation and management of excess histones during the cell cycle. Front. Biosci..

[12] Yang T., Li Y., Su B. (2022). Understanding the multiple roles of extracellular histones in mediating endothelial dysfunction. J. Am. Soc. Nephrol..

[13] Yokoyama Y., Ito T., Yasuda T., Furubeppu H., Kamikokuryo C., Yamada S., Maruyama I., Kakihana Y. (2019). Circulating histone h3 levels in septic patients are associated with coagulopathy, multiple organ failure, and death: A single-center observational study. Thromb. J..

[14] Jiang L., Li Y., Du H., Qin Z., Su B. (2021). Effect of anticoagulant administration on the mortality of hospitalized patients with COVID-19: An updated systematic review and meta-analysis. Front. Med..

[15] Ligi D., Lo Sasso B., Della Franca C., Giglio R.V., Agnello L., Ciaccio M., Mannello F. (2023). Monocyte distribution width alterations and cytokine storm are modulated by circulating histones. Clin. Chem. Lab. Med..

[16] Zhou X., Jin J., Lv T., Song Y. (2024). A narrative review: The role of nets in acute respiratory distress syndrome/acute lung injury. Int. J. Mol. Sci..

[17] Zhang X., Li X. (2022). The role of histones and heparin in sepsis: A review. J. Intensive Care Med..

[18] Lv S., Zhang S., Zuo J., Liang S., Yang J., Wang J., Wei D. (2023). Progress in preparation and properties of chitosan-based hydrogels. Int. J. Biol. Macromol..

[19] Reay S.L., Jackson E.L., Salthouse D., Ferreira A.M., Hilkens C.M.U., Novakovic K. (2023). Effective endotoxin removal from chitosan that preserves chemical structure and improves compatibility with immune cells. Polymers.

[20] Cheng Y., Chen Y., Song T., Jiang L., He Y., Li Y., Zhao W., Su B. (2025). Simultaneous removal of endotoxin and circulating histones by heparin-grafted chitosan-cellulose composite microspheres for multitargeted hemoperfusion in septic blood. Biomacromolecules.

[21] Kr Ner F., Hubbuch J. (2013). Systematic generation of buffer systems for ph gradient ion exchange chromatography and their application. J. Chromatogr. A.

[22] Sharkey B., Pudi S., Wallace Moyer I., Zhong L., Prinz B., Baruah H., Lynaugh H., Kumar S., Wittrup K.D., Nett J.H. (2017). Purification of common light chain igg-like bispecific antibodies using highly linear ph gradients. Mabs.

[23] Dellinger R.P., Bagshaw S.M., Antonelli M., Foster D.M., Klein D.J., Marshall J.C., Palevsky P.M., Weisberg L.S., Schorr C.A., Trzeciak S. (2018). Effect of targeted polymyxin b hemoperfusion on 28-day mortality in patients with septic shock and elevated endotoxin level: The euphrates randomized clinical trial. JAMA.

[24] Klein D.J., Foster D., Walker P.M., Bagshaw S.M., Mekonnen H., Antonelli M. (2018). Polymyxin b hemoperfusion in endotoxemic septic shock patients without extreme endotoxemia: A post hoc analysis of the euphrates trial. Intensive Care Med..

[25] Li Y., Sun P., Chang K., Yang M., Deng N., Chen S., Su B. (2022). Effect of continuous renal replacement therapy with the oxiris hemofilter on critically ill patients: A narrative review. J. Clin. Med..

[26] Zang S., Chen Q., Zhang Y., Xu L., Chen J. (2022). Comparison of the clinical effectiveness of an69-oxiris versus an69-st filter in septic patients: A single-centre study. Blood Purif..

[27] Guan M., Wang H., Tang X., Zhao Y., Wang F., Zhang L., Fu P. (2022). Continuous renal replacement therapy with adsorbing filter oxiris in acute kidney injury with septic shock: A retrospective observational study. Front. Med..

[28] Wong E.T., Ong V., Remani D., Wong W., Haroon S., Lau T., Nyeo H., Mukhopadhyay A., Tan B., Chua H. (2021). Filter life and safety of heparin-grafted membrane for continuous renal replacement therapy—A randomized controlled trial. Semin. Dial..

[29] Scharf C., Liebchen U., Paal M., Irlbeck M., Zoller M., Schroeder I. (2021). Blood purification with a cytokine adsorber for the elimination of myoglobin in critically ill patients with severe rhabdomyolysis. Crit. Care.

[30] Honoré P.M., Matson J.R. (2004). Extracorporeal removal for sepsis: Acting at the tissue level--the beginning of a new era for this treatment modality in septic shock. Crit. Care Med..

[31] König C., Röhr A.C., Frey O.R., Brinkmann A., Roberts J.A., Wichmann D., Braune S., Kluge S., Nierhaus A. (2019). In vitro removal of anti-infective agents by a novel cytokine adsorbent system. Int. J. Artif. Organs.

[32] Harm S., Falkenhagen D., Hartmann J. (2014). Pore size—A key property for selective toxin removal in blood purification. Int. J. Artif. Organs.

[33] Scully M., Levi M. (2019). How we manage haemostasis during sepsis. Br. J. Haematol..

[34] Mu L., Wu L., Wu S., Ye Q., Zhong Z. (2024). Progress in chitin/chitosan and their derivatives for biomedical applications: Where we stand. Carbohydr. Polym..

[35] Li X., Dong W., Nalin A.P., Wang Y., Pan P., Xu B., Zhang Y., Tun S., Zhang J., Wang L.-S. (2018). The natural product chitosan enhances the anti-tumor activity of natural killer cells by activating dendritic cells. Oncoimmunology.

[36] Cassinelli G., Naggi A. (2016). Old and new applications of non-anticoagulant heparin. Int. J. Cardiol..

[37] Wat J.M., Audette M.C., Kingdom J.C. (2018). Molecular actions of heparin and their implications in preventing pre-eclampsia. J. Thromb. Haemost..

[38] Simard J.M., Schreibman D., Aldrich E.F., Stallmeyer B., Le B., James R.F., Beaty N. (2010). Unfractionated heparin: Multitargeted therapy for delayed neurological deficits induced by subarachnoid hemorrhage. Neurocrit Care.

[39] Litov L., Petkov P., Rangelov M., Ilieva N., Lilkova E., Todorova N., Krachmarova E., Malinova K., Gospodinov A., Hristova R. (2021). Molecular mechanism of the anti-inflammatory action of heparin. Int. J. Mol. Sci..

[40] Song Y., Wu Y., Ding F., Li S., Shen Y., Yang B., Tang X., Ren L., Deng L., Jin X. (2024). The preventive and therapeutic effects of acute and severe inflammatory disorders with heparin and heparinoid. Biomolecules.

[41] Li L., Ling Y., Huang M., Yin T., Gou S., Zhan N., Xiong J.-X., Wu H.-S., Yang Z.-Y., Wang C.-Y. (2015). Heparin inhibits the inflammatory response induced by lps and hmgb1 by blocking the binding of hmgb1 to the surface of macrophages. Cytokine.

[42] Lelubre C., Vincent J. (2018). Mechanisms and treatment of organ failure in sepsis. Nat. Rev. Nephrol..

[43] Patel B.V., Lee T.M.L., O’Dea K. (2023). Clustering circulating histones in sepsis. Am. J. Respir. Crit. Care Med..

[44] Xu J., Zhang X., Pelayo R., Monestier M., Ammollo C.T., Semeraro F., Taylor F.B., Esmon N.L., Lupu F., Esmon C.T. (2009). Extracellular histones are major mediators of death in sepsis. Nat. Med..

[45] Talbert P.B., Henikoff S. (2021). Histone variants at a glance. J. Cell Sci..

[46] Zhang H., Wang Y., Qu M., Li W., Wu D., Cata J.P., Miao C. (2023). Neutrophil, neutrophil extracellular traps and endothelial cell dysfunction in sepsis. Clin. Transl. Med..

[47] Evans L., Rhodes A., Alhazzani W., Antonelli M., Coopersmith C.M., French C., Machado F.R., Mcintyre L., Ostermann M., Prescott H.C. (2021). Surviving sepsis campaign: International guidelines for management of sepsis and septic shock 2021. Crit Care Med.

